# Trends in Incidence of Stroke and Transition of Stroke Subtypes in Rural Tianjin China: A Population-Based Study from 1992 to 2012

**DOI:** 10.1371/journal.pone.0139461

**Published:** 2015-10-01

**Authors:** Bin Li, Yongzhong Lou, Hongfei Gu, Xue Long, Tao Wang, Jian Wei, Jinghua Wang, Jun Tu, Xianjia Ning

**Affiliations:** 1 Department of Neurology, Tianjin Haibin People’s Hospital, Tianjin, China; 2 Department of Neurology, Tianjin Medical University General Hospital & Department of Epidemiology, Tianjin Neurological Institute, Tianjin, China; University of Florida, UNITED STATES

## Abstract

**Objectives:**

The incidence of ischemic stroke has increased and that of hemorrhagic stroke has decreased in urban China; however, the trends in rural areas are unknown. We aimed to explore the secular trends in incidence and transition of stroke subtypes among rural Chinese.

**Methods:**

This was a population-based stroke surveillance through the Tianjin Brain Study. A total of 14,538 residents in a township of Ji County in Tianjin, China participated in the study since 1985. We investigated the age-standardized stroke incidence (sex-specific, type-specific, and age-specific), the annual proportion of change in the incidence of stroke, and the proportion of intracerebral hemorrhage in the periods 1992–1998, 1999–2005, and 2006–2012, because the neuroimaging technique was available since 1992 in this area.

**Results:**

The age-standardized incidence per 100,000 person-years increased significantly for both intracerebral hemorrhage (37.8 in 1992–1998, 46.5 in 1999–2005, and 76.5 in 2006–2012) and ischemic stroke (83.9 in 1992–1998, 135.3 in 1999–2005, and 238.0 in 2006–2012). The age-standardized incidence of first-ever stroke increased annually by 4.9% for intracerebral hemorrhage and by 7.3% for ischemic stroke. The greatest increase was observed in men aged 45–64 years for both stroke types (P < 0.001). The proportion of intracerebral hemorrhage was stable overall, increased among men aged 45–64 years, and decreased among men aged ≥65 years. The average age of intracerebral hemorrhage in men reduced by 7.5 years from 1992 to 2012.

**Conclusion:**

The age-standardized incidence of main stroke subtypes increased significantly in rural China over the past 21 years; the overall proportion of intracerebral hemorrhage was stable, but the incidence increased significantly among middle-aged men. These findings imply that it is crucial to control stroke risk factors in middle-aged men for stroke prevention in future decades.

## Introduction

Stroke is the leading cause of death and disability in both developed and developing countries worldwide [1–3]. The age-standardized incidence of stroke has declined over the last 30 years in developed countries [4–6]. Meanwhile, stroke incidence and the proportion of pathologic subtypes have changed significantly with aging of the worldwide population and the prevalence of stroke risk factors. In developed countries and certain developing countries, there has been an increase in the proportion of ischemic strokes (IS) and a decrease in the proportion of intracerebral hemorrhage (ICH) [7–9].

In China, stroke is the leading cause of death in rural areas but is the third most common cause of death in urban areas [10]. A study from an urban population in Beijing from 1984 to 2004 indicated that the incidence of ICH decreased while that of IS increased annually [11]. Identical trends were observed in another study from 3 cities in China [8]. However, there are currently no reports describing the long-term trends in the incidence of stroke subtypes among rural residents in China. The rural Chinese account for one-tenth of the world’s total population and have poor medical insurance, low education level, and low income. The previous studies have indicated that the incidence of first-ever stroke in rural Chinese increased rapidly in past twenty years [12,13]. However, the transition of stroke subtypes following increased incidence of stroke is unknown in rural China; therefore, the prevention of stroke in rural China is crucial to reduce stroke incidence in China.

The aim of this study was to explore the secular trends and epidemiological transition in incidence of first-ever stroke subtypes from 1992 to 2012 in a large rural population in Tianjin, northern China.

## Methods

### Study population

The study population was recruited to the Tianjin Brain Study, a population-based study on stroke incidence and mortality in a township of Ji County, Tianjin, China. The details of the study population were described in a previous publication [13–14]. Briefly, This study was conducted from a township of Ji County in Tianjin, China since 1985, which was chosen as the representative sample of rural residents in northern China to participate the national projects of stroke surveillence. The total population was 15,438 persons distributed within 18 administrative villages, and 95% of residents were low-income farmers. This population had a low income and low education level, and few participants were covered by national medical insurance before 2008 [12,15]; the population characteristics remained stable over the study period.

Stroke events and stroke-related deaths were monitored in this population since 1985. In this study, we analyzed the events of first-ever stroke from 1992, the year at which new diagnostic imaging techniques were first available.

The ethics committee of Tianjin Medical University General Hospital (TMUGH) approved the study, and written informed consent was obtained from each resident.

### Definition of stroke events

First-ever stroke was defined as the first occurrence of rapidly developing signs of focal neurologic disturbance of presumed vascular etiology lasting for >24 hours [16]. Main pathologic types of stroke included ICH and IS, defined as thrombotic brain infarction, cardioembolic stroke, or lacunar infarct. All patients with documented stroke experienced significant clinical symptoms and signs; silent strokes (diagnosed by imaging only) were excluded. Because of the low use of diagnostic imaging from 1992 to 1998, the neurologist from TMUGH identified stroke subtypes by using imaging information or clinical examination, depending on imaging availability.

### Network of stroke surveillance and quality control

Stroke events were reported as follows: local licensed village doctors reported initial stroke events to the community hospital within 24 hours of onset; doctors in the community hospital then visited patients’ homes to confirm stroke events within 72 hours. They reported confirmed stroke events (diagnosis by imaging) to TMUGH monthly, and the neurologist identified suspected cases (non-imaging) by interview as soon as possible.

To ensure accurate recording of stroke events, all participating doctors were annually trained on the predefined study protocol. The Quality Control Group consisted of senior epidemiologists and neurologists from the Department of Neurology, TMUGH. They conducted an omission survey annually by comparing multiple overlapping sources (hospital admissions register, local death register, and by interviewing patients’ relatives).

### Cardiovascular risk factors survey

The cardiovascular risk factors were examined in 1991 and 2011. The method of population sampling has been published [14]. Briefly, there were 2,196 (73%) participants in 1991 and 1939 (78%) participants in 2011, which aged 35–74 years without previous history of coronary heart disease or stroke, were involved in the survey. Detailed information included demographical information, and risk factors. The physical examinations included measurement of blood pressure, body height, and weight. Hypertension was defined as systolic blood pressure (SBP) ≥ 140 mmHg and/or diastolic blood pressure (DBP) ≥ 90 mmHg or receiving medications for hypertension. Diabetes was defined as self-reported previous diabetes history. Body mass index (BMI) was calculated as weight in kilograms divided by the square of height in meters, and obesity was defined as BMI ≥ 28 kg/m^2^. Current smoking was dined as smoking at least 1 cigarette/daily more than 1 year, and alcohol consumption was defined as drinking alcohol at least weekly more than 1 year. Fasting glucose (FG), total cholesterol (TC), triglycerides (TG) in the serum were tested in the central laboratory of the Tianjin Neurological Institute. Because of limited funding, there were 1092 participants aged 35–64 years in 1991 and 1939 individuals aged 35–74 years in 2011.

### Statistical analysis

In this study, the incidence of ICH and IS were analyzed separately for 1992–1998, 1999–2005, and 2006–2012; computed tomography (CT) has been widely used since 1999, and magnetic resonance imaging (MRI) became available in 2006 in this area. The age-standardized incidences were calculated directly using the world standard population in 10 age-groups: <35, 35–39, 40–44, 45–49, 50–54, 55–59, 60–64, 65–69, 70–74, and ≥75 years [17]. The age-specific incidence of first-ever stroke was assessed by three group: < 45 years, 45–64 years, and ≥65 years. Trends in age-standardized incidence of stroke were expressed as the annual percentage of change, using the regression model log (r_t_) = a + bt, where log denotes the natural logarithm and t is the year. The trend b was estimated from ordinary regression [11], and 100b represented the estimated annual percentage of change of incidence. In addition, we performed a chi-square test to analysis the changes in levels of cardiovascular disease risk factors between 1991 and 2011. Analyses included all patients, with or without available imaging, because there were no significant differences in demographic characteristics between these patients (Table 1). Statistical significance was defined as P < 0.05. SPSS version 15.0 for Windows (SPSS Inc., Chicago, IL, USA) was used for the analyses [18].

**Table 1:** The demographic characteristics and proportion of related risk factors between patients diagnosis with and without imaging in the Tianjin Brain Study.

Characteristics	1992–1998		1999–2005		2006–2012	
	Imaging	Clinical	Imaging	Clinical	Imaging	Clinical
Male, n (%)	51 (63.0)	62 (63.9)	114 (59.7)	43 (63.2)	233 (58.5)	39 (53.4)
Age of onset, years, means(SE)	60.7 (1.1)	71.8 (1.0)*	63.0 (0.8)	71.7 (1.4)*	63.3 (0.6)	76.3 (1.1)*
Age group, n (%)						
<45	7 (8.6)	1 (1.0)*	12 (6.3)	1 (1.5)*	25 (6.3)	0*
45–64	44 (54.3)	19 (19.6)*	79 (41.4)	11 (16.2)*	208 (52.3)	11 (15.1)*
≥65	30 (37.0)	77 (79.4)*	100 (52.4)	56 (82.4)*	165 (41.5)	62 (84.9)*
Subtype, n (%)						
Intracerebral hemorrhage	26 (49.1)	27 (50.9)	52 (27.2)	11 (16.2)*	101 (25.4)	7 (9.6)*
Ischemic stroke	55 (45.5)	66 (54.5)*	138 (72.8)	52 (83.8)*	292 (74.6)	61 (90.4)*
Death within 30 days, n (%)	15 (23.1)	25 (28.1)	24 (21.2)	13 (23.6)	41 (35.7)	13 (26.5)
Hypertension, n (%)	67 (82.7)	74 (76.3)	176 (92.1)	54 (79.4)*	371 (93.2)	63 (86.3)*
Diabetes, n (%)	2 (2.5)	1 (1.0)	15 (7.9)	2 (2.9)	39 (9.8)	8 (11.0)
Smoking, n (%)	39 (48.1)	43 (44.3)	88 (46.1)	33 (48.5)	185 (46.5)	26 (35.6)
Alcohol consumption, n (%)	19 (23.5)	22 (22.3)	45 (23.6)	13 (19.1)	129 (32.4)	12 (16.4)*

* indicated P<0.05 in Chi-Square test for trend between the study periods.

## Results

### Characteristics of first-ever stroke patients

During 304,260 person-years of follow-up, we identified 908 patients with first-ever stroke, 224 (24.7%) patients with ICH, and 664 (73.1%) patients with IS, 7 (0.8%) with SAH, and 13 (1.4%) with USD. The age of first-ever stroke (ICH) in men decreased by 7.5 years (69.2 vs. 61.7; P = 0.003) over 21 years, but this trend was not observed in women. The proportion of diagnoses by imaging (CT/MRI) improved from 46.6% to 85.4% during the study period (P < 0.001) (Table 2).

**Table 2:** The descriptive characteristics of patients with first-ever stroke by gender and period in Tianjin Brain Study.

Characteristics	Men			Women			Total		
	1992–1998	1999–2005	2006–2012	1992–1998	1999–2005	2006–2012	1992–1998	1999–2005	2006–2012
Person-year:	53644	52073	52344	50162	48224	47813	103806	100297	100157
Education level: year, mean(SE)									
	1.0(0.2)	3.1(0.3)	4.4(0.2)*	0.9(0.2)	1.5(0.2)	2.8(0.1)*	1.0(0.2)	2.5(0.2)	3.8(0.2)*
Age of onset, years, means(SE)									
ICH^†^	69.2(1.9)	62.9(1.9)	61.7(1.5)*	60.9(2.2)	63.6(2.3)	62.4(1.9)	66.4(1.5)	63.2(1.5)	62.0(1.2)
IS^†^	67.3(1.2)	66.4(1.0)	65.6(0.8)	65.6(1.8)	65.0(1.5)	67.0(1.0)	66.6(1.0)	65.9(0.9)	66.2(0.6)
Total	67.9(1.0)	65.6(0.9)	64.6(0.7)*	64.3(1.5)	64.6(1.3)	65.9(0.7)	66.6(0.8)	65.2(0.7)	65.2(0.6)
Diagnosis by CT/MRI, n(%)									
ICH^†^	17(48.6)	30(85.7)	61(91.0)*	9 (50.0)	23(76.7)	45(97.8)*	26(49.1)	53(81.5)	106(93.8)*
IS^†^	34(45.3)	84(70.6)	172(84.7)*	21(45.7)	54(76.1)	120(80.0)*	55(45.5)	138(72.6)	292(82.7)*
Total	51(46.4)	114(74.0)	233(86.3)*	30(46.9)	77(76.2)	165(84.2)*	81(46.6)	191(74.9)	398(85.4)*

*indicated P<0.05 in Chi-Square test between the study periods.

^†^ICH = intracerebral hemorrhage, IS = ischemic stroke.

### Age-standardized incidence and relative risk of first-ever stroke

The age-standardized incidence of ICH and IS increased steadily over the past 21 years in both sexes (Table 3). The relative risk (RR) of first-ever stroke, both ICH and IS, increased with time in both sexes compared to 1992–1998; the RR for ICH in 2006–2012 was 1.9 (95% confidence interval [CI] 1.3–2.9) overall (P = 0.001), 1.7 (95% CI 1.0–2.6) in men (P = 0.030), and 2.7 (95% CI 1.3–5.4) in women (P = 0.004). The corresponding RR for IS was 2.8 (95% CI 2.2–3.6) overall, 2.6 (95% CI 2.0–3.6) in men, and 3.2 (95% CI 2.1–4.8) in women (P < 0.0001,Table 4). The age-specific incidence of ICH and IS increased significantly among people aged 45–64 years, with ICH increasing by 2.9 times overall, 6.8 times in men, and 1.4 times in women, and IS increasing by 2.5 times overall, 2.4 times in men, and 2.7 times in women (P < 0.0001). Nevertheless, the incidence of first-ever stroke among participants aged ≥65 years increased significantly for IS, but appeared unvaried for ICH in all patients (Table 3), increased significantly for ICH in patients with neuroimaging diagnosis (S1 Table).

**Table 3:** The age-standardized age-specific and sex-specific incidence of intracerebral hemorrhage and ischemic stroke in the Tianjin Brain Study (1/100000 person-year).

Age group(years)	Intracerebral hemorrhage			Ischemic stroke		
	1992–1998	1999–2005	2006–2012	1992–1998	1999–2005	2006–2012
Men(SE)						
Total	53.6(34.0, 73.2)	48.6(29.2, 68.0)	89.6(63.9, 109.4)*	107.0(79.4, 632.1)	173.4(137.7, 209.1)	280.2(234.9, 325.5)*
<45	6.2(0, 14.2)	3.2(0, 9.5)	22.5(5.8, 39.2)*	6.2(0, 14.8)	15.9(2.0, 29.8)	22.5(5.8, 39.2)
45–64	30.9(3.9, 57.9)	112.2(58.9, 165.5)	235.1(159.4, 310.8)*	173.3(109.2, 237.4)	250.7(171.1, 330.3)	591.0(471.2, 710.8)*
≥65	533.5(336.3, 730.7)	311.5(163.7, 459.3)	381.3(218.4, 544.2)	857.5(608.0, 1107.0)	1392.7(1081.8, 1703.6)	1870.3(1512.4, 2228.2)*
Women(SE)						
Total	22.9(9.6, 36.2)	41.0(23.0, 59.0)	57.9(36.3, 79.5)*	62.1(40.3, 83.9)	103.0(74.4, 131.6)	197.9(158.1, 237.7)*
<45	0	11.3(0, 24.0)	15.6(0, 30.9)*	14.6(0, 28.9)	15.1(0, 29.8)	23.5(4.7, 42.3)
45–64	63.5(26.1, 100.9)	50.0(15.3, 84.7)	138.0(81.6, 194.4)*	103.9(55.9, 151.9)	150.1(90.1, 210.1)	384.0(290.1, 477.9)*
≥65	127.7(33.2, 222.1)	297.0(156.1, 437.9)	286.4(146.3, 426.5)	437.8(263.0, 612.6)	751.4(527.6, 975.2)	1432.2(1120.6, 1743.8)*
Overall(SE)						
Total	37.8(26.0, 49.6)	44.7(31.6, 57.8)	72.8(56.1, 89.5)*	83.9(66.3, 101.5)	135.3(112.6, 158.0)	238.0(207.8, 268.2)*
<45	3.4(0, 8.1)	6.9(0.3, 13.6)	19.4(7.8, 31.0)*	10.1(2.1, 18.1)	15.5(5.3, 25.7)	22.9(10.4, 35.4)
45–64	47.8(24.5, 71.1)	80.3(48.9, 111.7)	185.2(138.4, 232.0)*	137.4(97.8, 177.0)	199.1(149.5, 248.7)	484.6(408.9, 560.3)*
≥65	326.2(218.4, 434.0)	301.4(200.3, 402.5)	333.5(226.3, 440.7)	643.1(491.8, 794.4)	1055.0(866.4,1243.6)	1649.7(1412.7, 1886.7)*

* P<0.05 in Chi-Square test for trend between the study periods.

**Table 4:** The related risk (95%CI) of main pathological stroke types incidence by sex and age in Tianjin Brain Study.

Age Group	Intracerebral hemorrhage			Ischemic Stroke		
	1992–1998	1999–2005	2006–2012	1992–1998	1999–2005	2006–2012
Men:						
<45	1.0	0.5 (0.1,5.6)	3.6 (0.7,17.5)	1.0	2.6 (0.1,13.2)	3.6 (0.7,17.5)
45–64	1.0	3.6 (1.3,9.8)*	7.8 (3.1,19.9)*	1.0	1.5 (0.9,2.4)	3.4 (2.2,5.2)*
≥65	1.0	0.6 (0.3,1.5)	0.7 (0.4,1.3)	1.0	1.6 (1.1,2.4)*	2.2 (1.5,5.2)*
Total	1.0	0.9 (0.5,1.5)	1.7 (1.0,2.6)*	1.0	1.6 (1.2,2.3)*	2.6 (2.0,3.6)*
Women:						
<45	—	1.0	1.4 (0.3,6.2)	1.0	0.7 (0.2,3.0)	1.6 (0.5,5.7)
45–64	1.0	0.9 (0.3,2.2)	2.4 (1.2,5.0)*	1.0	1.4 (0.8,2.7)	3.7 (2.2,6.3)*
≥65	1.0	2.3 (0.9,5.6)	2.2 (0.9,5.5)	1.0	1.7 (1.0,2.8)*	3.3 (2.1,5.2)*
Total	1.0	1.9 (0.9,3.9)	2.7 (1.3,5.4)*	1.0	1.7 (1.1,2.6)*	3.2 (2.1,4.8)*
overall:						
<45	1.0	2.1 (0.4,11.2)	5.8 (1.3,26.1)*	1.0	1.5 (0.5,4.3)	2.3 (0.9,6.0)
45–64	1.0	1.7 (0.9,3.1)	3.9 (2.2,6.7)*	1.0	1.4 (1.0,2.1)*	3.5 (2.5,4.9)*
≥65	1.0	0.9 (0.6,1.5)	1.0 (0.6,1.6)	1.0	1.6 (1.2,2.2)*	2.6 (2.0,3.4)*
Total	1.0	1.2 (0.8,1.8)	1.9 (1.3,2.9)*	1.0	1.6 (1.2,2.1)*	2.8 (2.2,3.6)*

* indicated P<0.05 comparing with the study period of 1992–1998.

### Proportion of ICH and IS by gender and age

The proportion of ICH among all stroke events remained stable overall: 29.8%, 24.3%, and 22.9% in 1992–1998, 1999–2005, and 2006–2012, respectively. However, the proportion of ICH by age group increased significantly among those aged 45–64 years: 34.0% in 1992–1998, 39.7% in 1999–2005, and 55.6% in 2006–2012 (P = 0.002). The corresponding proportions among men were 14.3%, 48.6%, and 56.9%, respectively (P < 0.0001). In contrast, the proportion of ICH decreased among those aged ≥65 years: 66.0% in 1992–1998, 54.0% in 1999–2005, and 34.3% in 2006–2012 in both sexes. The corresponding proportions among men were 80.0%, 48.6%, and 32.3%, respectively (P < 0.0001). No corresponding changes were found in women (Table 5 and S2 Table).

**Table 5:** The proportion of main stroke type by sex and age (95% CI).

Group	1992–1998	1999–2005	2006–2012
Men:			
Total	31.0(22.6, 39.4)	22.3(15.8, 28.8)	23.9(18.8, 29.0)
<45	50.0 (0, 99)	16.7 (0, 46.5)	50.0 (23.8, 76.2)
45–64	15.2 (2.9, 27.4)	29.8 (17.9, 41.7)	28.2 (20.5, 35.9)*
≥65	36.8 (26.0, 47.7)	18.1 (10.3, 25.9)	16.5 (10.1, 23.0)*
Women:			
Total	27.7(16.7, 38.7)	27.5(18.9, 36.1)	21.1(15.4, 26.8)
<45	0	42.9 (6.2, 79.5)	36.4 (7.9, 64.8)
45–64	36.7 (19.4, 53.9)	24.2 (9.6, 38.9)	26.1 (17.0, 35.3)
≥65	22.6 (7.9, 37.3)	27.4 (16.3, 38.5)	16.0 (8.8, 23.2)
Overall:			
Total	29.8(23.1, 36.5)	24.3(19.0, 29.6)	22.9(19.2, 26.6)
<45	25.0 (0, 55.0)	30.8 (5.7, 55.9)	44.0 (24.5, 63.5)
45–64	25.4 (14.7, 36.1)	27.8 (18.5, 37.0)	27.4 (21.5, 33.3)*
≥65	32.7 (23.8, 41.6)	21.8 (15.3, 28.3)	16.3 (11.5, 21.1)*

* indicated P<0.05 in Chi-Square test for trend between the study periods.

### Annual changes in incidence of ICH and IS

**Fig 1:**
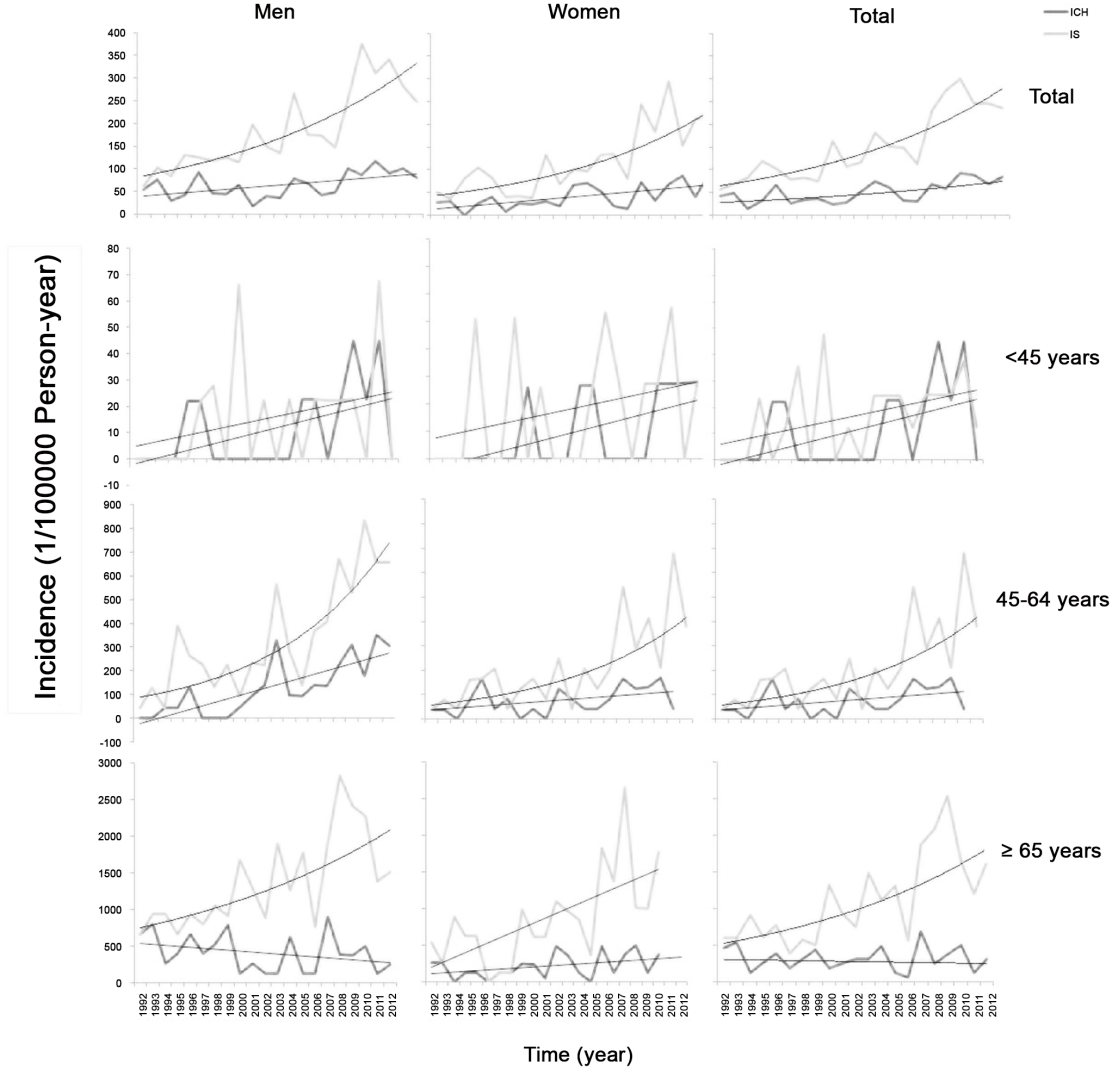
Age-standardized incidence of intracerebral hemorrhage and ischemic stroke by gender and age groups from 1992 to 2012 (1/100,000 person-years). The age-standardized incidence increased annually by 4.9% for intracerebral hemorrhage (ICH) and by 7.3% for ischemic stroke (IS). The incidence in those aged 45–64 years increased annually by 11.8% for ICH and by 10.5% for IS (P < 0.001). The incidence of first-ever stroke in those aged ≥65 years remained stable for ICH but increased by 6.2% in IS.

Fig 1 depicts the age-standardized incidence of ICH and IS by gender and age. The age-standardized incidence of ICH increased annually by 4.9% (3.7% in men, 5.1% in women), and IS increased annually by 7.3% (6.8% in men, 8.3% in women). The incidence of ICH among those aged 45–64 years increased by 11.8% annually overall and by 9.8% in men; the incidence of IS in those aged 45–64 years increased annually by 10.5% overall, by 10.7% in men, and by 9.9% in women (P < 0.001). The incidence of first-ever stroke among those aged ≥65 years remained stable for ICH, but increased for IS (6.2% overall, 5.1% in men, 7.4% in women annually).

### Differences in cardiovascular risk factors between 1991 and 2011

The prevalence of hypertension, diabetes, obesity, and alcohol consumption was significantly higher in 2011 than in 1991, all P<0.05; the prevalence of smoking, in contrast, decreased from 23.6% in 1991 to 19.0% in 2011. The prevalence of obesity significantly increased in all age groups. The prevalence of risk factors was obvious elevated in young group, except for diabetes; but among mid-life, the prevalence of all risk factors significantly increased, current smoking was decreased. The dramatic different trend was found in old group, the prevalence of hypertension and alcohol consumption stained unchanged, but the rate of current smoking significantly decreased (Table 6).

**Table 6:** The prevalence of cardiovascular risk factors in study population between 1991 and 2011.

Risk factors	Men		Women		Total	
	1991	2011	1991	2011	1991	2011
n (%)	1032	865	1164	1074	2196	1939
Hypertension, %(SE)	38.7 (1.1)	50.3 (1.7)*	41.1 (1.4)	53.6 (1.5)*	39.9 (1.1)	51.7 (1.1)*
<45 years	23.5(2.1)	31.2 (3.2)*	23.7(1.8)	39.9 (3.9)*	23.6(1.4)	34.9 (2.4)*
45~64 years	42.0 (2.3)	58.9 (2.3)*	45.4 (2.2)	57.6 (1.8)*	43.7 (1.6)	58.1 (1.4)*
≥65 years	66.1(3.9)	68.4 (3.3)	71.4(3.6)	74.1 (3.1)	68.6(2.6)	71.2 (2.2)
Obesity, %(SE)	2.6 (3.2)	17.8 (1.3)*	8.4 (2.8)	20.9 (2.7)*	5.7 (2.1)	19.5 (2.0)*
<45 years	1.7 (0.7)	16.5 (2.6)*	7.2 (1.1)	18.1 (3.1)*	4.8 (0.7)	17.2 (1.9)*
45~64 years	3.1 (2.8)	18.4 (1.8)*	9.8 (1.3)	22.3 (1.6)*	6.6 (0.8)	20.8 (1.2)*
≥65 years	3.5 (1.4)	18.5 (2.7)*	6.7 (2.0)	21.2 (2.9)*	5.0 (1.2)	20.2 (2.0)*
Diabetes*, % (SE)	1.4 (3.2)	3.5 (3.2)*	3.5 (2.9)	3.9 (2.5)*	2.5 (2.2)	3.7 (2.0)*
<45 years	1.0(0.5)	0.9(0.7)	2.7(0.7)	0.6(0.6)	2.0(0.05)	0.8(0.05)
45~64 years	2.0(0.7)	4.9(1.0)*	4.9(1.0)	5.8(0.9)	3.5(0.6)	5.4(0.7)*
≥65 years	0.6(0.6)	4.4(1.4)*	0.6(0.6)	8.3(1.9)*	0.6(0.4)	6.3(1.2)*
Smoking*, %(SE)	46.0 (2.2)	36.8 (2.7)*	3.7 (2.9)	4.8 (3.0)*	23.6 (1.9)	19.0 (2.0)*
<45 years	50.9(2.5)	63.5(3.3)*	1.3(0.5)	0.6(0.6)	23.0(1.4)	36.3(2.5)*
45~64 years	45.9(2.3)	37.6(6.9)*	5.9(1.1)	4.5(0.8)	25.3(1.4)	17.4(1.1)*
≥65 years	34.9(3.7)	7.3(1.8)*	4.5(1.7)	8.7(2.0)	20.3(2.2)	8.0(1.3)*
Alcohol drinking*, %(SE)	18.9 (2.8)	31.6 (2.8)*	0.3 (1.5)	4.5 (3.0)*	9.1 (2.0)	16.6 (2.1)*
<45 years	21.1(2.0)	47.4(3.4)*	0.2(0.2)	0.6(0.6)	9.3(1.0)	30.1(2.4)*
45~64 years	18.0(1.8)	31.4(2.2)*	0.4(0.3)	4.1(0.7)*	9.0(0.9)	14.7(1.0)*
≥65 years	16.0(2.8)	10.2(2.1)	0.6(0.6)	8.7(2.0)*	8.6(1.6)	9.5(1.4)
FG mmol/L, mean(SD)	4.8(1.5)	5.2(1.7)*	4.6(0.9)	5.3(1.8)*	4.7(1.2)	5.3(1.8)*
TC mmol/L, mean(SD)	4.3(0.9)	4.6(1.1)*	4.3(1.0)	4.8(1.2)*	4.3(1.0)	4.7(1.2)*
TG mmol/L, mean(SD)	1.3(0.3)	1.4 (1.0)*	1.3(0.3)	1.6(1.0)*	1.3(0.3)	1.5(1.03)*

* indicated P<0.05 in Chi-Square test for comparing between two study periods.

## Discussion

This is the first up-to-date, long-term report of epidemiological transition in the incidence and proportion of main pathologic stroke subtypes among rural residents in China. While the incidence of stroke has decreased in developed countries [7, 19–21], it has rapidly increased in developing countries, especially China [8,11].

The incidence of ICH and IS decreased in Japan from 1991 to 2005 [18]. From 1983 to 1997, the incidence of IS in Finland decreased, but the incidence of ICH remained stable [22]. Similarly, in the Greater Cincinnati/Northern Kentucky Stroke Study, the incidence of IS decreased in the white population, and ICH remained unchanged in the white and black population between 1993 and 2005 [23]. The Oxford Vascular Study reported a decrease of more than 50% in incidence of ICH between 1981 and 2004 [7]. Nonetheless, different trends have emerged in China. Some studies have reported an upward trend in the incidence of IS and a downward trend in the incidence of ICH in urban China [8,11]. However, the incidence of IS increased significantly and that of ICH was unchanged in Hong Kong and Changsha [24,25]. Previous studies reported that patterns of stroke subtypes in Chinese populations were rapidly adopting a Western pattern [8,26,27]. In the last century, the proportion of ICH was higher in Chinese than in white populations [23,28–31].

In this study, we observed upward trends in the incidence of both ICH and IS from 1992 to 2012. The age-standardized incidence increased annually by 4.9% for ICH and by 7.3% for IS. Simultaneously, the prevalence of hypertension, obesity, diabetes, high fasting glucose, and alcohol consumption in this study population increased significantly by 29.6%, 253.6%, 95.8%, 524.1%, and 82.4%, respectively, from 1991 to 2011. These may explain the increase in overall and age-specific incidence of both ICH and IS. Moreover, lower awareness of risk factors and poor medical sources may be the causes of the conversed trends in the incidence of ICH and IS between urban and rural in China.

The proportion of ICH in this study tended to remain stable overall, but increased significantly in participants aged 45–64 years, especially in men. In contrast, the proportion of ICH decreased in those aged ≥65 years.

The previous studies have suggested that hypertension, diabetes, smoking, alcohol, and obesity have been evidenced to be the risk factor for ICH [32–39]. The increased prevalence of hypertension in those aged 35–64 years and the increased prevalence of alcohol consumption in men over the past 21 years may partly explain the greater proportion of ICH in participants aged 45–64 years. The stable prevalence of hypertension and alcohol consumption, and the decreased rate of current smoking in old group may partly explain the decreased proportion of ICH in elders. Moreover, the lower control rate of hypertension in this low-income population (0.7% in 1991, 12% in 2011) may explain the predominance of ICH in this study [14].

Rapid economic development in China may be a potential explanation for the upward trend in prevalence rates of CRFs in our study. Over the past 21 years, there has been an extensive shift in the level of agricultural mechanization, resulting in a significant decrease in the need for rural laborers. Urbanization should also be considered as a rational reason to explain higher prevalence of CVD and its risk factors in rural population [40]. Though China has the largest population in developing countries with an unbalanced development throughout the whole country, the westernization of lifestyles is accelerated not only among urban residents but also among rural population. Documents from the Ministry of Health of the People's Republic of China showed that the energy ratio obtained from cereal decreased 15.3%, but the energy ratio obtained from animals and fat increased by 87.1% and 48.9%, respectively, among rural residents from 1992 to 2002 [41].

An increased incidence of stroke has been reported among those of lower SES in the previous studies [42–44]. A meta-analysis of 17 studies published between 1980 and 2008 demonstrated an increased incidence of stroke in those of lower SES (pooled hazard ratio, 1.67 [1.46–1.91]) [45]. Higher rates of both ischemic and hemorrhagic strokes were found in men and women from lower SES (using area-based deprivation index) in a study conducted in Italy [42]. Consistent with these studies, we found the increased incidence both in ICH and in IS in this low-income, low-education population.

The limitation of this study is, first, the lower proportion of diagnoses verified by CT/MRI (46% during 1992–1998). However, there was no significant difference in percentage of diagnosis by neuroimaging between ICH and IS (P = 0.661), and all stroke events without imaging data were verified by senior neurologists from TMUGH. Second, this study population is from a township in northern China, which does not represent the national population. The prospective study design and the long follow-up period overcome these limitations. At least 100,000 person-years of observations during all 3 study periods fulfill the criteria for a high-quality stroke incidence study [46].

## Conclusions

Our study is the first to provide up-to-date secular trends in the incidence of pathological stroke subtypes in a low-income population during a 21-year period in rural China. The incidence of ICH and IS increased, while the proportion of ICH increased in young and middle aged men and decreased in the elderly population. The increased prevalence of alcohol consumption and hypertension, along with a low hypertension control rate, may explain the findings of the present study. Thus, it is crucial to control these risk factors in middle-aged men to prevent stroke in future decades in China.

## Supporting Information

S1 Table The age-standardized and age-specific incidence of stroke diagnosis by imaging (1/100000 person-year). (DOCX)

S2 Table The age-specific proportion of intracerebral hemorrhage diagnosis by imaging (95% CI). (DOCX)
