# Supplementary material for: Trends in Incidence of Stroke and Transition of Stroke Subtypes in Rural Tianjin China: A Population-Based Study from 1992 to 2012
Source: PLoS One. 2015 Oct 1;10(10):e0139461. doi: 10.1371/journal.pone.0139461 (PMC4591354; doi:10.1371/journal.pone.0139461)
Supplement: S1 Table — (DOCX) [file pone.0139461.s001.docx]

**S1 Table.** The age-standardized and age-specific incidence of stroke diagnosis by imaging (1/100000 person-year).

| Age group  (years) | Intracerebral hemorrhage | | |  | Ischemic Stroke | | |  |
| --- | --- | --- | --- | --- | --- | --- | --- | --- |
|  | 1992-1998 | 1999-2005 | 2006-2012 |  | 1992-1998 | 1999-2005 | 2006-2012 |  |
| Men(95%CI) | | | | | | | | |
| Total | 23.9 (10.8,37.0) | 42.0 (24.4,59.6) | 84.6 (59.7,109.5)^*^ |  | 46.0 (27.8,64.1) | 120.8 (90.9,150.6) | 234.6 (193.2,276.1)^*^ |  |
| <45 | 3.1 (0,9.2) | 3.2 (0,9.4) | 22.5 (5.8,39.2)^*^ |  | 6.2 (0,14.8) | 12.7 (0.2,25.2) | 22.5 (5.8,39.2) |  |
| 45-64 | 30.9 (3.8,58.0) | 99.0 (48.9,149.0) | 222.4 (148.8,296.0)^*^ |  | 111.4 (60.0,162.8) | 217.7 (143.5,291.9) | 572.0 (454.1,689.8)^*^ |  |
| ≥65 | 209.6 (170.9,248.1) | 256.6 (122.3,390.8) | 345.0 (190.1,499.9) |  | 266.8 (127.2,406.3) | 861.3 (616.1,1106.5) | 1361.9 (1055.8,166.80)^*^ |  |
| Women(95%CI) | | | | | | | | |
| Total | 10.9 (1.8,20.1) | 33.7 (17.4,50.1) | 61.2 (39.0,83.4)^*^ |  | 27.5 (13.0,42.0) | 79.3 (54.2,104.4) | 159.8 (124.0,195.6)^*^ |  |
| <45 | 0 | 11.3 (0,24.1) | 19.6 (2.4,36.7)^*^ |  | 14.6 (0.3,28.9) | 15.1 (0.3,29.9) | 23.5 (4.7,42.2) |  |
| 45-64 | 46.2 (14.1,78.3) | 50.0 (15.4,84.7) | 144.0 (86.4,201.6)^*^ |  | 75.0 (34.3,115.8) | 143.9 (85.1,202.6) | 354.0 (263.8,444.2)^*^ |  |
| ≥65 | 18.2(6.42,30.1) | 209.7 (91.2,328.2) | 286.4 (146.3,426.6)^*^ |  | 73.0 (1.5,144.5) | 471.8 (294.2,649.3) | 984.6 (725.7,1243.5)^*^ |  |
| Overall(95%CI) | | | | | | | | |
| Total | 17.5 (9.5,25.6) | 38.1 (26.0,50.2) | 72.1 (55.5,88.7)^*^ |  | 36.5 (24.9,48.1) | 97.6 (116.9) | 196.1 (168.7,223.5)^*^ |  |
| <45 | 1.7 (0,5.0) | 6.9 (0.1,13.6) | 21.2 (9.2,33.2)^*^ |  | 10.1 (2.0,18.1) | 13.8 (4.2,23.4) | 21.2 (9.2,33.2) |  |
| 45-64 | 38.8 (17.7,59.9) | 73.8 (43.7,104.0) | 182.1 (135.7,228.5)^*^ |  | 92.6 (0,193.6) | 179.8 (132.8,226.9) | 459.9 (386.2,533.5)^*^ |  |
| ≥65 | 111.8 (48.6,175.1) | 230.5 (142.1,319.0) | 315.5 (211.1,420.0)^*^ |  | 167.8 (90.3,245.2) | 656.0 (507.1,805.0) | 1171.9 (972.2,1371.6)^*^ |  |

* P<0.05 in Chi-Square test for trend between the study periods.
