# Supplementary material for: Trends in Incidence of Stroke and Transition of Stroke Subtypes in Rural Tianjin China: A Population-Based Study from 1992 to 2012
Source: PLoS One. 2015 Oct 1;10(10):e0139461. doi: 10.1371/journal.pone.0139461 (PMC4591354; doi:10.1371/journal.pone.0139461)
Supplement: S2 Table — (DOCX) [file pone.0139461.s002.docx]

**S2 Table.** The age-specific proportion of intracerebral hemorrhage diagnosis by imaging (95% CI).

| Group | 1992-1998 | 1999-2005 | 2006-2012 | P |
| --- | --- | --- | --- | --- |
| Men (95%CI): | | | |  |
| Total | 33.3(20.4, 46.2) | 26.3(18.3, 34.3) | 26.2(20.5, 31.9) | 0.393 |
| <45 | 33.3 (0, 86.7) | 20.0 (0, 55.1) | 50.0 (23.8, 76.2) | 0.379 |
| 45-64 | 21.7 (4.9, 38.6) | 31.3 (18.1, 44.4) | 28.0 (20.1, 35.9) | 0.754 |
| ≥65 | 44.0 (24.5, 63.5) | 23.0 (12.4, 33.5) | 20.2 (12.1, 28.3) | 0.032 |
| Women (95%CI): | | | |  |
| Total | 30.0(13.5, 46.5) | 29.9(19.7, 40.1) | 27.3(20.4, 34.2) | 0.664 |
| <45 | 0 | 42.9 (6.2, 79.5) | 45.5 (16.0, 74.9) | 0.163 |
| 45-64 | 38.1 (17.3, 58.9) | 25.8 (10.4, 41.2) | 28.9 (19.2, 74.9) | 0.552 |
| ≥65 | 20.0 (0, 55.1) | 30.8 (16.3, 45.3) | 22.5 (12.8, 32.3) | 0.542 |
| Total (95%CI): | | | |  |
| Total | 32.1(21.9, 42.3) | 27.7(21.4, 34.0) | 26.6(22.3, 30.9) | 0.353 |
| <45 | 14.3 (0, 40.2) | 33.3 (6.7, 60.0) | 48.0 (28.4, 67.6) | 0.098 |
| 45-64 | 29.5 (16.1, 43.0) | 29.1 (19.1, 39.1) | 28.4 (22.2, 34.5) | 0.0857 |
| ≥65 | 40.0 (22.5, 45.8) | 26.0 (17.4, 34.6) | 21.2 (15.0, 27.4) | <0.001 |
